# ﻿Diversity of jumping spiders (Araneae, Salticidae) in the Jinggangshan National Nature Reserve, Jiangxi, China

**DOI:** 10.3897/zookeys.1239.140810

**Published:** 2025-05-23

**Authors:** Bin-lu Liu, Yan-bin Yao, Zi-han Cai, Zhong-jing Wang, Ke-ke Liu

**Affiliations:** 1 Key Laboratory of Jiangxi Province for Biological Invasion and Biosecurity, Jinggangshan University, Ji’an 343009, Jiangxi, China Jinggangshan University Ji’an China; 2 Jinshan College of Fujian Agriculture and Forestry University, Fuzhou 350007, Fujian, China Jinshan College of Fujian Agriculture and Forestry University Fuzhou China

**Keywords:** Distribution, new record, new species, survey, taxonomy

## Abstract

A list of 94 salticid species of 48 genera collected from Jinggangshan National Nature Reserve in Jiangxi Province is provided. Four new salticid species are diagnosed, described, and illustrated: *Charippuswangzuo* K. Liu, **sp. nov.**, *Cytaeahezizhen* K. Liu, **sp. nov.**, *Orientattuschushu* K. Liu, **sp. nov.** and *Yaginumanisyuanwencai* K. Liu, **sp. nov.**. Previously unknown females of *Eupoapengi* Liu, 2021, and *Yaginumaellalobata* Peng, Tso & Li, 2002 are described for the first time. In contrast, the data also shows that there are many nature reserves with a high percentage of unknown spider species in need of further intensive and extensive survey. Hence, our study will make a significant contribution to our understanding of species diversity and the importance of the reserve.

## ﻿Introduction

In the past, Chinese arachnologists paid more attention to the spider species from the southwest of the country (Himalayan and Hengduan mountains, and the Yunnan-Guizhou Plateau) (Li and Lin 2016; Li 2020). Adding Guangxi, Hunan, and Hainan to those provinces, they have all become a biodiversity hotspot for researchers. Recent updates of the spider species distributions of 72 families of spiders demonstrated the existence of 5875 species of spiders under 894 genera (The Biodiversity Committee of Chinese Academy of Sciences, 2023). More than half of the spider species have been discovered in these six areas. However, species richness of spiders in non-hotspots, such as Jiangxi, Fujian, and Guangdong (Jiang et al. 2023) Provinces have not been given enough attention based on their rich biological resources. Recently, in Jiangxi and Fujian, a large number of spider taxa have been discovered by the team, such as Phrurolithidae (Liu et al. 2020a, b, 2021, 2022a, 2023), Thomisidae (Liu et al. 2017, 2022c; Li et al. 2023), Gnaphosidae (Liu et al. 2022b), and Leptonetidae (Liu et al. 2024). Despite this, there are still many more spider diversities to be discovered using a systematic faunal survey program in these provinces.

The Salticidae Blackwall, 1841, or jumping spiders, is the largest family, with a global distribution, comprising 6634 extant species belonging to 677 genera (WSC 2024). They occur in a wide variety of habitats and inhabit terrestrial ecosystems globally (Peng 2020; WSC 2024), including most island environments, although absent from continental Antarctica and regions with permanent polar ice sheets. Diverse predatory strategies have evolved in the family, including araneophagy, aggressive mimicry, myrmecophagy, and prey-specific prey-catching behavior based on their dazzling predatory behaviors (Jackson and Pollard 1996), and they are able to survive in all habitats and microhabitats due to a long evolutionary process (Marc et al. 1999). The combination of narrow spatial niches occupied by most species and diverse microhabitats within a site would explain the high diversity of jumping spiders (Argañaraz et al. 2017). However, to the best of our knowledge, there is no publication on the diversity of jumping spiders in China (e.g., Wang et al. 2023b).

At present, more than one-tenth of all world’s salticid species were recorded from China, including 143 genera and 744 species (WSC 2024). Although many species have been reported in the past five years (WSC 2024), there are still many poorly known salticids and other species (Liu et al. 2022d) from this country with unusual morphological characteristics. Yunnan Province currently represents the most species-rich region in China, with approximately 250 documented species (Wang et al. 2024a). In contrast, Jiangxi Province remains relatively understudied, where only 27 species have been recorded to date (Li and Lin 2016). To fill the knowledge gap, we studied a large collection from the Jinggang Mountain Nature Reserve in this province.

Jinggang Mountain is located in the middle section of Luoxiao Mountains, a mountain system formed by several northeast-southwest trending mountains, and has complex and diverse geology and landforms with nearly 2000 meters of elevation difference. There are approximately 3200 species of vascular plants which provide a favorable environment for animals in this mountain (Chen et al. 2012). Currently, more than 2700 insect species, 270 bird species, 60 mammal species, 60 reptile species, 40 amphibian species, and 40 fish species are known from this mountain (Chen et al. 2012). However, the spider fauna was mainly neglected in the past except the Phrurolithidae, Thomisidae, and Anyphaenidae (Liu et al. 2020a, 2022c; Wang et al. 2024b).

The aims of the present paper are to report findings of 94 species belonging to 48 genera, to provide detailed descriptions of four new species, to provide descriptions of previously unknown females of two species, and to reveal some of the species diversity occurring in this mountain.

## ﻿Materials and methods

Specimens were examined using a SZ6100 stereomicroscope. Both male and female copulatory organs were dissected and examined in 80% ethanol using an Olympus CX43 compound microscope with a KUY NICE CCD camera. Epigynes were cleared with pancreatin solution (Álvarez-Padilla and Hormiga 2007). Images were captured with a KUY NICE CCD mounted on Zeiss Axio Scope A1 compound microscope and assembled using Helicon Focus v. 6.7.1 (Helicon Soft Ltd.) image stacking software. For SEM photographs, the specimens were kept under natural dry conditions, coated with gold with a small ion-sputtering apparatus ETD-2000, and photographed with a Zeiss EVO LS15 scanning electron microscope. All the images were processed in Adobe Photoshop CS 5. The measurements were taken using a stereomicroscope (AxioVision SE64 Rel. 4.8.3) and are given in millimeters. The body lengths of all specimens exclude the chelicerae and spinnerets. Leg measurements are given as total length (femur, patella, tibia, metatarsus, tarsus). The distribution map was generated with ArcGIS v. 10.8 (ESRI Inc.). All specimens are deposited in Animal Specimen Museum, Life Science of College, Jinggangshan University (**ASM-JGSU**).

Terminology of male and female copulatory organs follows Wang (2022) and Wang et al. (2023b). The abbreviations used in the figures and text are as follows: **ALE** = anterior lateral eye, **AME** = anterior median eye, **At** = atrium, **CD** = copulatory duct, **CO** = copulatory opening, **d** = dorsal, **Em** = embolus, **FD** = fertilization duct, **GA** = glandular appendage, **Ho** = hood, **MOA** = median ocular area, **MS** = median septum, **p** = prolateral, **PL** = posterior lobe, **PLE** = posterior lateral eye, **PME** = posterior median eye, **r** = retrolateral, **RTA** = retrolateral tibial apophysis, **SD** = sperm duct, **Spe** = spermatheca, **TA** = tegular apophysis, **TF** = tegular flap, **v** = ventral, **VTA** = ventral tibial apophysis.

## ﻿Results

A total of 652 adults belonging to 94 species and 48 genera were recorded, of which 77 species (82%) were strictly endemic in Jiangxi Province. One quarter of the species belongs to four genera: *Phintella* Strand, 1906 (13 species), *Myrmarachne* MacLeay, 1839 (7 species), *Synagelides* Strand in Bosenberg and Strand 1906 (6 species), and *Ptocasius* Simon, 1885 (5 species). Ten species are probably new to science, but only four species are described as new in this work, due to the fact that these represent paired species with complete morphological characters, whereas the remaining six are based solely on unisex specimens lacking diagnostic features for formal description. Two species are supplemented by the previously unknown other sex in the taxonomic section. In addition, among the 48 genera recorded, 36 genera including *Asemonea* O. Pickard-Cambridge, 1869, *Bianor* Peckham & Peckham, 1886, *Charippus* Thorell, 1895, *Cheliceroides* Żabka, 1985, *Chinattus* Logunov, 1999, *Chinophrys* Zhang & Maddison, 2012, *Cytaea* Keyserling, 1882, *Epeus* Peckham & Peckham, 1886, *Euochin* Prószyński, 2018, *Gelotia* Thorell, 1890, *Harmochirus* Simon, 1885, *Hasarina* Schenkel, 1963, *Hasarius* Simon, 1871, *Icius* Simon, 1876, *Irura* Peckham & Peckham, 1901, *Lechia* Żabka, 1985, *Myrmarachne* MacLeay, 1839, *Neon* Simon, 1876, *Nigorella* Wesolowska & Tomasiewicz, 2008, *Nungia* Zabka, 1985, *Onomastus* Simon, 1900, *Orientattus* Caleb, 2020, *Pancorius* Simon, 1902, *Portia* Karsch, 1878, *Ptocasius* Simon, 1885, *Rhene* Thorell, 1869, *Sibianor* Logunov, 2001, *Siler* Simon, 1889, *Spartaeus* Thorell, 1891, *Stertinius* Simon, 1890, *Telamonia* Thorell, 1887, *Thiania* C. L. Koch, 1846, *Thyene* Simon, 1885, *Toxeus* C. L. Koch, 1846, *Vailimia* Kammerer, 2006, and *Yaginumanis* Wanless, 1984 are newly recorded in Jiangxi Province. Notably, the genus *Phintella* dominated the assemblage with 13 species, accounting for 13.8% of the total recorded species. This is followed by *Myrmarachne* with seven species and *Ptocasius* with five species. Two species, *Phintellabifurcilinea* (Bösenberg & Strand, 1906) and *Ptocasiusstrupifer* Simon, 1901, had the highest number of individuals collected in this study. At present, the known jumping spider fauna of Jiangxi Province increases to 110 species in 56 genera. These findings highlight the reserve as a hotspot for salticid diversity in subtropical China. Future studies correlating microhabitat parameters (e.g., vegetation structure, elevation gradients) with salticid distribution patterns could further elucidate mechanisms underlying this observed diversity. Conservation prioritization for habitat types supporting high endemicity (e.g., forest understories hosting undescribed taxa) is recommended (Table 1).

**Table 1.** List of Salticidae species recorded in Jiangxi Province. Genera and species recorded for the first time are marked with an asterisk (*).

Genus	Species	No. of ♂♂	No. of ♀♀	Total
*Aelurillus* Simon, 1884	*A.m-nigrum* (Kulczyński, 1891)	1♂		1
*Asemonea* O. Pickard-Cambridge, 1869*	*A.sichuanensis* (Song & Chai, 1992)*		2♀	2
*Bianor* Peckham & Peckham, 1886*	B. *balius* Thorell, 1890*	1♂	4♀	5
*Carrhotus* Thorell, 1891	*C.sannio* (Thorell, 1877)	1♂	3♀	4
	*C.viduus* (C.L. Koch, 1846)*	3♂	3♀	6
	*C.xanthogramma* (Latreille, 1819)	1♂	2♀	3
*Charippus* Thorell, 1895*	*C.wangzuo* sp. nov.*	1♂	3♀	4
*Cheliceroides* Żabka, 1985*	*C.longipalpis* Żabka, 1985*	6♂	3♀	9
*Chinattus* Logunov, 1999*	*C.tibialis* (Żabka, 1985)*	3♂	5♀	8
*Chinophrys* Zhang & Maddison, 2012*	*C.pengi* Zhang & Maddison, 2012*	1♂	2♀	3
*Cytaea* Keyserling, 1882*	*C.hezizhen* sp. nov.*	2♂	4♀	6
*Epeus* Peckham & Peckham, 1886*	*E.bicuspidatus* (Song, Gu & Chen, 1988)*		2♀	2
	*E.guangxi* Peng & Li, 2002*	2♂		2
	*E.glorius* Żabka, 1985*	1♂	8♀	9
*Euochin* Prószyński, 2018*	*E.atrata* (Song & Chai, 1992)*	1♂	3♀	4
	*E.* sp. nov.*	1♂		1
*Eupoa* Zabka, 1985	*E.pengi* Liu, 2021	3♂	3♀	6
*Evarcha* Simon, 1902	*E.albaria* (L. Koch, 1878)	10♂	14♀	24
	*E.fasciata* Seo, 1992*	2♂		2
	*E.flavocincta* (C.L. Koch, 1846)*		9♀	9
	*E.pococki* Zabka, 1985*	2♂	1♀	3
*Gelotia* Thorell, 1890*	*G.syringopalpis* Wanless, 1984*	1♂		1
*Harmochirus* Simon, 1885*	*H.brachiatus* (Thorell, 1877)*	5♂	6♀	11
*Hasarina* Schenkel, 1963*	*H.contortospinosa* Schenkel, 1963*		1♀	1
*Hasarius* Simon, 1871*	*H.adansoni* (Auodouin, 1826)*		1♀	1
*Icius* Simon, 1876*	*I.bilobus* Yang & Tang, 1996*	1♂		1
*Irura* Peckham & Peckham, 1901*	*I.longiochelicera* (Peng & Yin, 1991)*	3♂	3♀	6
*Lechia* Żabka, 1985*	*L.squamata* Żabka, 1985*		2♀	2
*Lokina* Yu, Maddison & Zhang, 2023	*L.nyuewa* Yu & Zhang, 2023	1♂	1♀	2
*Menemerus* Simon, 1868	*M.brachygnathus* (Thorell, 1887)	1♂		1
*Myrmarachne* MacLeay, 1839*	*M.angusta* (Thorell, 1877)*		1♀	1
	*M.formicaria* (De Geer, 1778)*		5♀	5
	*M.gisti* Fox, 1937*	10♂	4♀	14
	*M.inermichelis* Bösenberg & Strand, 1906*	2♂	1♀	3
	*M.kuwagata* Yaginuma, 1967*	17♂		17
	*M.hamata* Wang, Mi & Peng, 2023*	1♂		1
	*M.* sp. nov.*		1♀	1
*Neon* Simon, 1876*	*N.minutus* Zabka, 1985*	11♂	9♀	20
*Nigorella* Wesolowska & Tomasiewicz, 2008*	*N.hirticeps* (Song & Chai, 1992)*	2♂		1
*Nungia* Zabka, 1985*	*N.epigynalis* Żabka, 1985*	2♂	5♀	7
*Onomastus* Simon, 1900*	*O.chenae* Lin & Li, 2020*		1♀	1
*Orientattus* Caleb, 2020*	*O.chushu* sp. nov.*	2♂	4♀	6
*Orienticius* Prószyński, 2016	*O.vulpes* (Grube, 1861)	2♂	2♀	4
*Pancorius* Simon, 1902*	*P.crassipes* (Karsch, 1881)*	1♂	1♀	2
	*P.magnus* Żabka, 1985*		2♀	2
*Phintella* Strand, 1906	*P.abnormis* (Bösenberg & Strand, 1906)*	1♂		1
	*P.aequipeiformis* Zabka, 1985*	8♂		8
	*P.arcuata* Huang, Wang & Peng, 2015*	3♂	2♀	5
	*P.banna* Wang & Li, 2020*		3♀	3
	*P.bifurcilinea* (Bösenberg & Strand, 1906)*	33♂	38♀	71
	*P.cavaleriei* (Schenkel, 1963)		12♀	12
	*P.fanjingshan* Li, Wang, Zhang & Chen, 2019*		1♀	1
	*P.linea* (Karsch, 1879)*	1♂	9♀	10
	*P.panda* Huang, Wang & Peng, 2015*		4♀	4
	*P.pulcherrima* Huang, Wang & Peng, 2015*	3♂	1♀	4
	*P.wulingensis* Huang, Wang & Peng, 2015*	1♂	6♀	7
	*P.* sp. nov. 1*		1♀	1
	*P.* sp. nov. 2*		1♀	1
*Phintelloides* Kanesharatnam & Benjamin, 2019	*P.versicolor* (C.L. Koch, 1846)	10♂	23♀	33
*Plexippus* C.L. Koch, 1846	*P.setipes* Karsch, 1879	5♂	8♀	13
*Portia* Karsch, 1878*	*P.heteroidea* Xie & Yin, 1991*	6♂	1♀	7
	*P.quei* Zabka, 1985*		5♀	5
	*P.wui* Peng & Li, 2002*	3♂		3
*Ptocasius* Simon, 1885*	*P.bulbosa* (Peng, Tang & Li, 2008)*	1♂		1
	*P.lushiensis* (Zhang & Zhu, 2007)*		9♀	9
	*P.montanus* (Żabka, 1981)*	2♂	7♀	9
	*P.strupifer* Simon, 1901*	20♂	46♀	66
	*P.wuermli* (Zabka, 1981)*	9♂	1♀	10
*Rhene* Thorell, 1869*	*R.albigera* (C.L. Koch, 1846)*		5♀	5
	*R.atrata* (Karsch, 1881)*	4♂	4♀	8
	*R.rubrigera* (Thorell, 1887)*	3♂	2♀	5
	*R.* sp. nov.*		2♀	2
*Sibianor* Logunov, 2001*	*S.aurocinctus* (Ohlert, 1865)*	2♂		2
	*S.latens* (Logunov, 1991)*		1♀	1
	*S.pullus* (Bösenberg & Strand, 1906)*	3♂	3♀	6
	*S.* sp. nov.*		3♀	3
*Siler* Simon, 1889*	*S.cupreus* Simon, 1889*	2♂	12♀	14
*Spartaeus* Thorell, 1891*	*S.platnicki* Song, Chen & Gong, 1991*	5♂	1♀	6
*Stertinius* Simon, 1890*	*S.donglinsiensis* Wang, Mi & Peng, 2023*		1♀	1
*Synagelides* Strand, 1906	*S.agoriformis* Strand, 1906*	5♂	9♀	14
	*S.annae* Bohdanowicz, 1979	3♂		3
	*S.jinggangshanensis* Liu, Chen, Xiao, Xu & Peng, 2017		1♀	1
	*S.lushanensis* Xie & Yin, 1990	1♂		1
	*S.tangi* Liu, Chen, Xiao, Xu & Peng, 2017	13♂	11♀	24
	*S.yinae* Liu, Chen, Xiao, Xu & Peng, 2017		2♀	2
*Telamonia* Thorell, 1887*	*T.caprina* (Simon, 1903)*		3♀	3
	*T.vlijmi* (Prószyński, 1984)*	2♂	1♀	3
*Thiania* C.L. Koch, 1846*	*T.suboppressa* Strand, 1907*	1♂	5♀	6
*Thyene* Simon, 1885*	*T.orientalis* Zabka, 1985*	1♂	1♀	2
	*T.yuxiensis* Xie & Peng, 1995*	1♂	1♀	2
*Toxeus* C.L. Koch, 1846*	*T.maxillosus* C.L. Koch, 1846*	2♂	2♀	4
*Vailimia* Kammerer, 2006*	*V.longitibia* Guo, Zhang & Zhu, 2011*		1♀	1
*Yaginumaella* Prószyński, 1979	*Y.lobata* Peng, Tso & Li, 2002	9♂	8♀	17
*Yaginumanis* Wanless, 1984*	*Y.yuanwencai* sp. nov.*	1♂	7♀	8

### ﻿Taxonomic account

**Family Salticidae Blackwall, 1841**

#### ﻿Genus *Charippus* Thorell, 1895

##### ﻿ Charippus wangzuo

Taxon classificationAnimaliaAraneaeSalticidae

K. Liu sp. nov.

26E904F6-240E-5F38-B01D-77A20753BECC

https://zoobank.org/E21A7290-25E0-4776-87B5-6E0F6877DEB9

**Figure 1.**
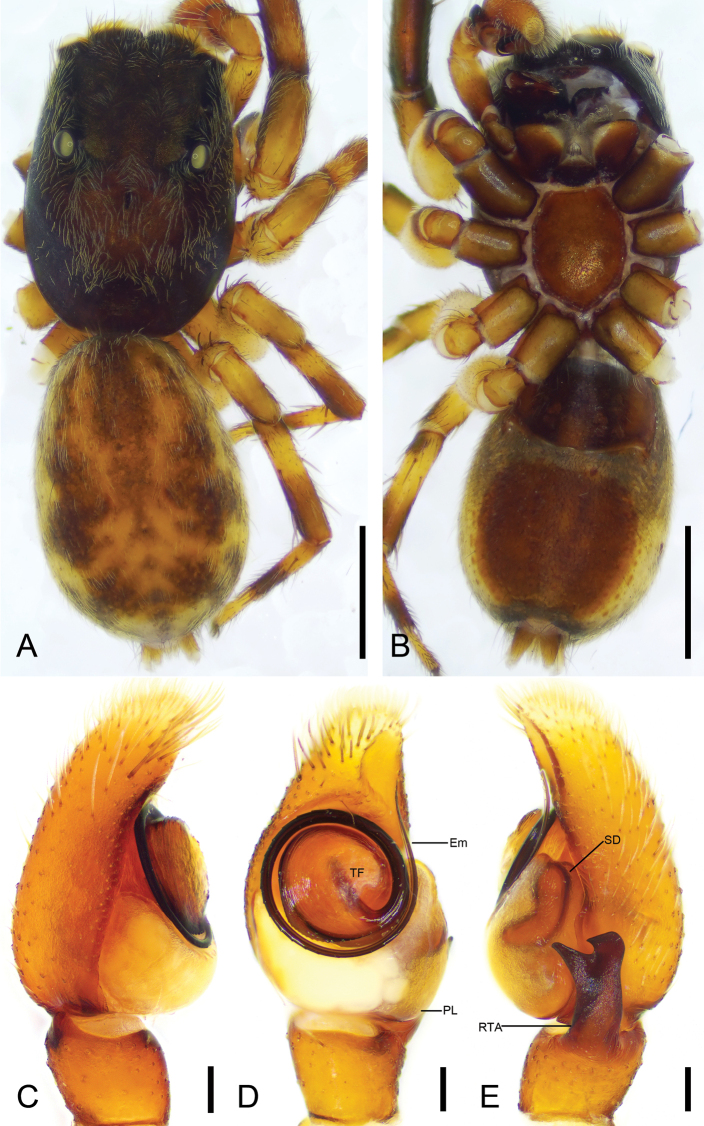
*Charippuswangzuo* sp. nov., male holotype **A** habitus, dorsal view **B** same, ventral view **C** palp, prolateral view **D** same, ventral view **E** same, retrolateral view. Abbreviations: Em – embolus, PL – posterior lobe, RTA – retrolateral tibial apophysis, SD – sperm duct, TF –tegular flap. Scale bars: 1 mm (**A**, **B**); 0.1 mm (**C**–**E**).

**Figure 2.**
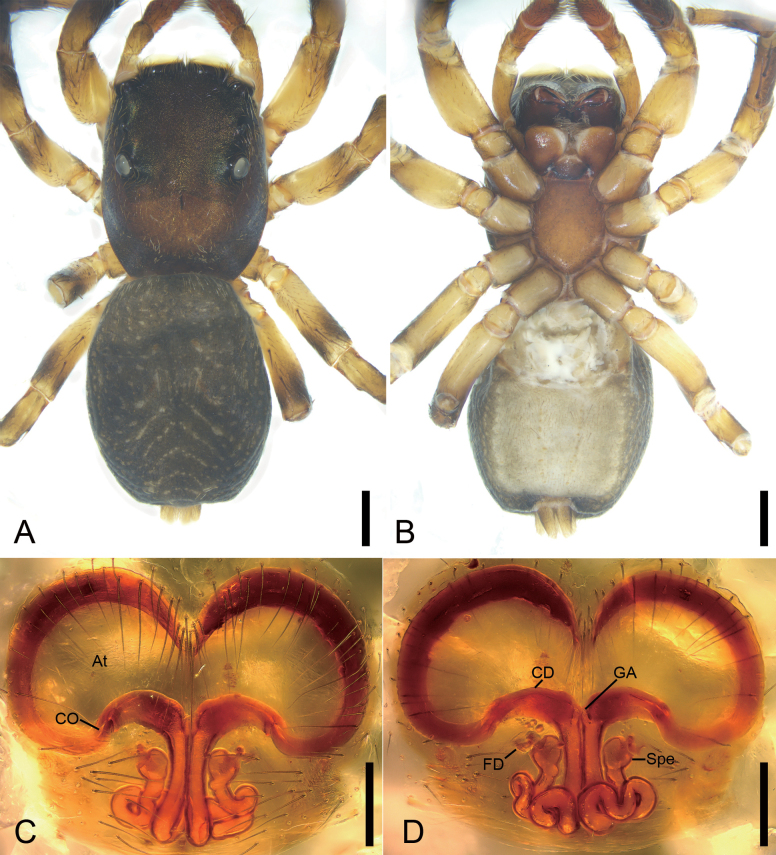
*Charippuswangzuo* sp. nov., female paratype **A** habitus, dorsal view **B** same, ventral view **C** epigyne, ventral view **D** same, dorsal view. Abbreviations: At – atrium, CD – copulatory duct, CO – copulatory opening, FD – fertilization duct, GA – glandular appendage, Spe – spermatheca. Scale bars: 0.5 mm (**A, B**); 0.2 mm (**C**, **D**).

Figs 1 , 2 , 12A Common name: 王佐查理蛛

###### Type material.

***Holotype*** ♂, China • Jiangxi Province: Ji’an County, Jinggangshan County Level City, Jinggang Mountain National Nature Reserve, Ciping Town, Zhufeng Scenic Spot, around Jingganghu, 26°31'58.8"N, 114°8'34.8"E, 723 m a.s.l., 1 August 2014, Feng-bo Zhang, Yong-hong Xiao, Chu-shu Xie, Huo-kai Wang, Jiao-rong Liu, Ze-yuan Meng and Ke-ke Liu leg. (Sal-028, ASM-JGSU). ***Paratypes***: • 1 ♀, Longtan Scenic Spot, 26°34'58.8"N, 114°8'6"E, 951 m a.s.l., 1 August 2014, Ke-ke Liu, Yu-bao Tang, Ze-yuan Meng, Xiao-ping Huang, Zhi-wu Chen, and Zhan-feng Wang leg., other data same as previous (Sal-035, ASM-JGSU); • Dalong Town, Yuantou Village, 26°37'40.8"N, 114°6'21.6"E, 906 m a.s.l., 5 April 2014, Ke-ke Liu, Yu-bao Tang, Ze-yuan Meng, Xiao-ping Huang, and Zhi-wu Chen leg., other data same as previous (Sal-035, ASM-JGSU); • Luofu Town, Xiangzhou Village, Baishuizhai, 26°36'10.8"N, 114°15'28.8"E, 375 m a.s.l., 29 May 2017, Ke-ke Liu, Ze-yuan Meng, Wen-jun Xie, and Zhi-wu Chen leg., other data same as previous (Sal-239, ASM-JGSU).

###### Diagnosis.

The male of this species is similar to that of *Charippusdenjii* Yu, Maddison & Zhang, 2022 (Yu et al. 2022: 166, figs 85, 86) in having the bifurcated retrolateral tibial apophysis and the anticlockwise spiraling embolus arising from 9 o’clock on tegulum and ending at ~ 12 o’clock on cymbial tip, but can be easily distinguished from it by the tibia with a thumb-like apex (vs pincer-like) (cf. Figs 1D, E, 12A and Yu et al. 2022: 166, figs 85, 86). The female also resembles that of *C.denjii* in having the heart-shaped atrium and the C-shaped anterior part of copulatory ducts, but can be separated from it by the posterior part of copulatory duct swerving for 7× (vs 6×) and the strongly swollen spermathecae far from (vs close to) the last turn (cf. Fig. 2C, D and Yu et al. 2022: 166, figs 90–93).

###### Description.

**Male (holotype).** Habitus (Fig. 1A, B). Total length 4.54. Carapace (Fig. 1A). Color yellow-brown; anterior part darker than posterior, lateral, and posterior margins covered by abundant, white, feathery setae, length 2.17, width 1.52. Eyes (Fig. 1A) diameters and interdistances: AME 0.35, ALE 0.23, PME 0.06, PLE 0.20; AME–AME 0.08, ALE−AME 0.07, PME–PME 1.03, PLE−PME 0.23, AME−PME 0.53, AME−PLE 0.82, ALE−ALE 0.86, PLE−PLE 0.89, ALE−PLE 0.65. MOA 0.75 long, front width 0.86, back width 1.11. Chelicerae (Fig. 1B) brown, with two retromarginal teeth and one large, bifurcated retromarginal tooth. Endites yellow slightly longer than wide. Labium yellow-brown, wider than long. Sternum shield-like, yellow-brown, longer than wide. Legs (Fig. 1A, B) yellow to brown, mottled; measurements: I 3.89 (1.23, 0.66, 0.98, 0.59, 0.43); II 3.57 (1.1, 0.66, 0.71, 0.69, 0.41); III 3.8 (1.15, 0.55, 0.74, 0.86, 0.5); IV 4.17 (1.12, 0.6, 0.95, 0.98, 0.52); spination: I Fe d 1-1-1, rt 0-0-1; Ti pv 1-1-1, rv 1-1-1; Met pv 1-0-1, rv 1-0-1; II Fe d 1-1-1, pr 0-0-1, rt 0-0-1; Pa pr 0-1-0; Ti pr 0-1-1, pv 1-0-1, rv 1-1-1; Met pr 1-0-1, pv 1-0-1, rv 1-0-1; III Fe d 1-1-1, rt 0-0-1, pr 0-0-1; Pa pr 0-1-0, rt 0-1-0; Ti pr 0-1-1, rt 0-1-1, pv 1-1-1, rv 1-0-1; Met pr 1-0-1, rt 1-0-1, pv 1-0-1, rv 1-0-1; IV Fe d 1-1-1, pr 0-0-1, rt 0-0-1; Pa pr 0-1-0, rt 0-1-0; Ti pr 1-1-1, rt 1-1-1, pv 1-0-1, rv 1-0-1; Met pr 1-1-1, rt 1-1-1, pv 1-0-1, rv 1-0-1. Abdomen (Fig. 1A, B) yellow to dark brown, anteriorly with abundant feathery and long setae dorsally; venter yellowish to brown, medially with two rows of yellow patches, 2.34 long, 1.59 wide.

***Palp*** (Figs 1C−E, 12A). Retrolateral tibial apophysis strongly sclerotized, spanner-like, longer than tibia. Sperm duct short, anterior part distinct. Posterior lobe swollen, slightly sclerotized and smooth. Tegular flap spiral, with thick apex, slightly sclerotized. Embolus curled, spiraling nearly 1 and ¾ coil, bearing dense denticles at outer edge, arising from 9 o’clock on tegulum and ending at ~ 12 o’clock near cymbial tip.

**Female (paratype).** Habitus (Fig. 2A, B). As in male, except as noted. Total length 4.18. Carapace (Fig. 2A) dark brown, length 2.14, width 1.50. Eye (Fig. 2A) diameters and interdistances: AME 0.42, ALE 0.25, PME 0.06, PLE 0.25; AME–AME 0.06, ALE−AME 0.13, PME–PME 1.04, PLE−PME 0.19, AME−PME 0.61, AME−PLE 0.83, ALE−ALE 0.88, PLE−PLE 0.94, ALE−PLE 0.59. MOA 1.15 long, front width 0.85, back width 1.24. Labium (Fig. 2B) red-brown. Legs measurements: I 2.64 (0.8, 0.71, 0.61, 0.31, 0.21); II 3.15 (0.95, 0.67, 0.59, 0.58, 0.36); III 3.5 (1.02, 0.6, 0.66, 0.81, 0.41); IV broken; spination (Fig. 2A, B): I Fe: d 1-1-0, pr0-0-2; Ti pv1-1-1, rv1-1-1; Met pv1-0-1, rv1-0-1; II Fe d1-1-2; Ti v1-1-1, rt0-0-1; Met pv1-0-1, rv1-0-1; III Fe d1-1-2, pr0-0-1; Pa pr1-1-0, rt1-1-0; Ti rv0-0-1, rt0-1-1; Met pr1-0-2, rt1-0-1, pv0-1-0, rv0-0-1, v0-0-2; IV Fe d1-1-0, rt0-0-2, pr0-0-1; Pa pr0-1-0, rt0-1-0; Ti and Met broken. Abdomen (Fig. 2A, B) ovoid, 2.23 long, 1.73 wide, dark brown, with six chevron-shaped stripes and many irregular yellow spots dorsally; venter grey.

***Epigyne*** (Fig. 2C, D). Atrium very large, covering nearly half epigynal area. Copulatory openings located at posterolateral part of atrium. Copulatory ducts very long, anterior part T-shaped extending, posterior part S-shaped in dorsal view. Glandular appendages clavate, slightly separated, directed anteriorly. Spermathecae oval, directed anteriorly. Fertilization ducts broad, curved.

###### Distribution.

Known only from the type locality in Jiangxi Province, China (Fig. 13).

###### Etymology.

The species is named in honor of Wang Zuo, one of the famous figures of the extremely hard and bitter struggle in Jinggangshan; noun in apposition.

#### ﻿Genus *Cytaea* Keyserling, 1882

##### ﻿ Cytaea hezizhen

Taxon classificationAnimaliaAraneaeSalticidae

K. Liu sp. nov.

A4F5BB65-342A-502E-B703-05E41F2B8B2B

https://zoobank.org/5947F1EA-15A3-4D77-84EA-CFF9C024DDC4

**Figure 3.**
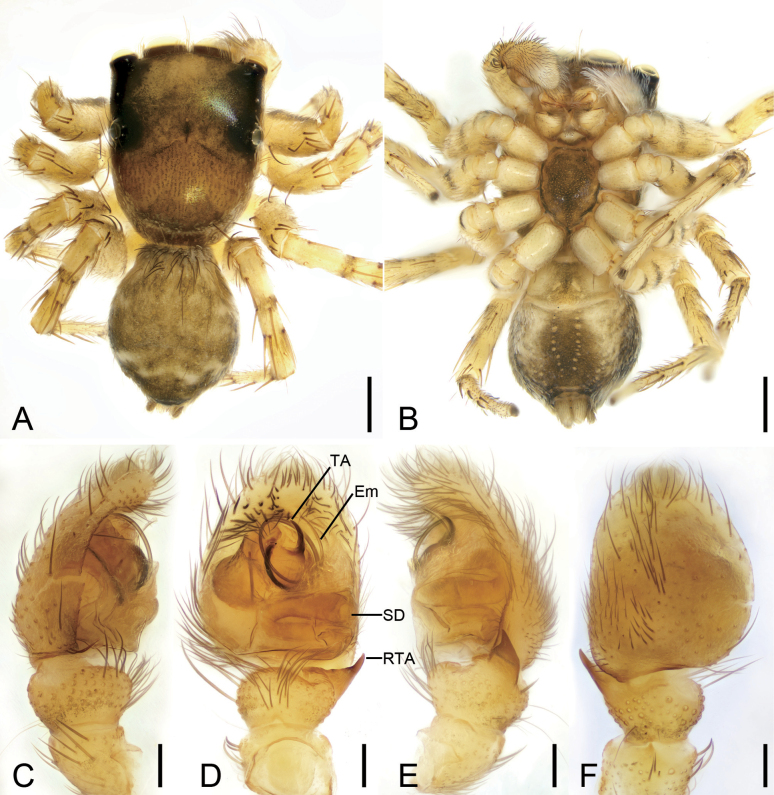
*Cytaeahezizhen* sp. nov., male holotype **A** habitus, dorsal view **B** same, ventral view **C** palp, prolateral view **D** same, ventral view **E** same, retrolateral view **F** same, dorsal view. Abbreviations: Em – embolus, RTA – retrolateral tibial apophysis, SD – sperm duct, TA – tegular apophysis. Scale bars: 0.5 mm (**A**, **B**); 0.1 mm (**C**–**F**).

**Figure 4.**
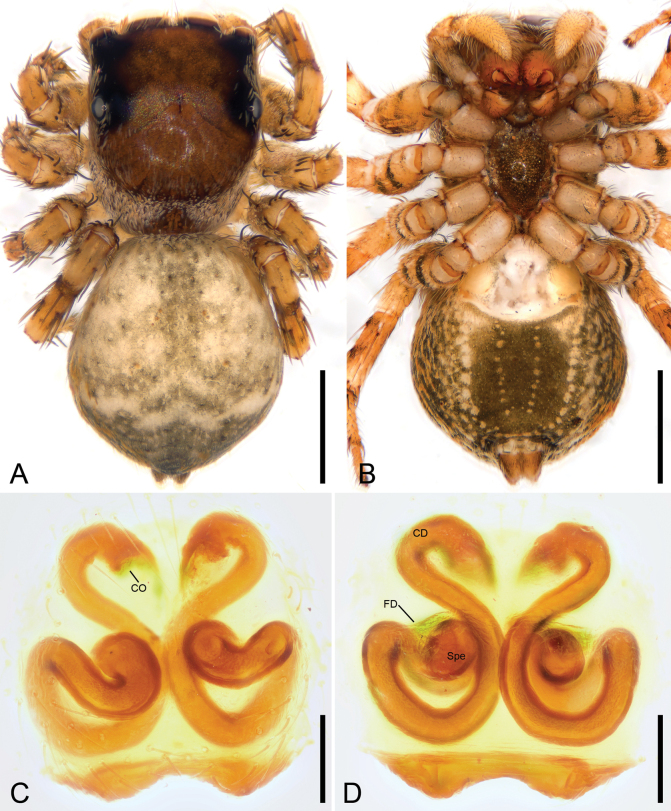
*Cytaeahezizhen* sp. nov., female paratype **A** habitus, dorsal view **B** same, ventral view **C** epigyne, ventral view **D** same, dorsal view. Abbreviations: CD – copulatory duct, CO – copulatory opening, FD – fertilization duct, Spe – spermatheca. Scale bars: 1 mm (**A, B**); 0.1 mm (**C**, **D**).

Figs 3 , 4 Common name: 贺子珍胞蛛

###### Type material.

***Holotype*** ♂, China • Jiangxi Province: Ji’an County, Jinggangshan County Level City, Longshi Town, Yuankou Village, 26°41'41.2"N, 113°57'21.6"E, 272 m a.s.l., l May 2015, Zhi-wu Chen, Sha Wu, Ze-yuan Meng, Shi-cong He, Yi-fan Zhao and Ke-ke Liu leg. (Sal-157, ASM-JGSU). ***Paratypes***: • 1 ♀, Xiazhuang Village, Zhushachong Forest Farm, 26°33'3.6"N, 114°11'20.4"E, 630 m a.s.l., 4 Oct 2014, Jian-yun Wen, Tian-ming Wang, Ze-yuan Meng, Lei Zhang and Ke-ke Liu leg., other data as same as holotype (Sal-157, ASM-JGSU).

###### Diagnosis.

The male of this species is similar to that of *Cytaeatongi* Wang & Li, 2020 in having the anticlockwise spiraling embolus with two circles partly covered by the membranous conductor, but can be distinguished from it by the tibia with short barb-like retrolateral apophysis (vs thick hook-shaped) and the small thin hook-shaped tegular apophysis on palpal tegulum (vs absent) (cf. Fig. 3C, D and Wang and Li 2020: 27, fig. 1B–D). Female resembles *C.maoming* Yu & Zhang, 2022 in having the long S-shaped copulatory ducts, but can be separated from it by the copulatory openings located anteriorly (vs medially), the pair of swan-like copulatory ducts with face-to-face format (vs back-to-back) and the spermathecae medially located at epigyne (vs subposteriorly) (cf. Fig. 4C, D and Yu and Zhang 2022: 5, fig. 5G–I).

###### Description.

**Male (holotype).** Habitus (Fig. 3A, B). Total length 3.75. Carapace (Fig. 3A). Color yellow-brown, anterior part darker than posterior, posteriorly with radial stripes, lateral and posterior margin covering by abundant white feathery setae, length 1.83, width 1.14. Eyes (Fig. 3A) diameters and interdistances: AME 0.29, ALE 0.26, PME 0.04, PLE 0.15; AME–AME 0.16, ALE−AME 0.13, PME–PME 1.19, PLE−PME 0.19, AME−PME 0.45, AME−PLE 0.66, ALE−ALE 0.98, PLE−PLE 1.16, ALE−PLE 0.59. MOA 0.58 long, front width 0.84, back width 1.30. Chelicerae (Fig. 3B) yellow, with three retromarginal teeth and one large bifurcated retromarginal tooth. Endites yellow brown, slightly longer than wide. Labium brown, wider than long. Sternum yellowish brown, longer than wide. Legs (Fig. 3A, B) yellow, mottled; measurements: I−II missing; III 2.83 (0.83, 0.56, 0.75, 0.34, 0.35); IV 2.56 (0.69, 0.74, 0.47, 0.39, 0.27); spination: III Fe d 1-1-1, rt 0-0-2; Pa pr 1-1-0, rt 1-1-0; Ti d 1-0-0, pr 1-1-0, rt 1-1-0, pv 1-0-1, rv 1-2-0; Met pr 1-0-2 rt 1-0-2 pv 1-0-0; IV Fe d 1-1-0, pr 0-0-2, rt 0-0-2; Pa pr 0-1-0 rt 0-1-0; Ti d 1-0-0, pr 1-2-0, rt 1-1-0, pv 1-0-1, rv 1-0-1; Met pr 1-1-1, rt 1-1-1, pv 1-0-0, rv 1-0-0. Abdomen (Fig. 3A, B) yellowish to dark yellowish brown, anteriorly with abundant feathery and long setae dorsally; venter yellow to brown, medially with two rows of yellow patches, 1.74 long, 1.36 wide.

***Palp*** (Fig. 3C−F). Retrolateral tibial apophysis short, barb-like, with broad base and an acute spine-like apex. Sperm duct S-shaped, located at retrolateral of tegulum. Tegulum with triangular membrane located medially on tegulum, anteriorly with a thin hook-shaped apophysis (tegular apophysis), directed prolaterally. Embolus whip-like, with two coils, spiraling anticlockwise, ending at ~ 12 o’clock.

**Female (paratype).** Habitus (Fig. 4A, B). As in male, except as noted below. Total length 3.91. Carapace (Fig. 4A). Color dark red-brown, length 1.80, width 1.64. Eyes (Fig. 4A) diameters and interdistances: AME 0.42, ALE 0.25, PME 0.05, PLE 0.22; AME–AME 0.13, ALE−AME 0.15, PME–PME 1.32, PLE−PME 0.22, AME−PME 0.60, AME−PLE 0.81, ALE−ALE 1.09, PLE−PLE 1.32, ALE−PLE 0.63. MOA 0.82 long, front width 0.88, back width 1.52. Chelicerae (Fig. 4B) red, with two retromarginal teeth and one large bifurcated retromarginal tooth. Labium red-brown, mottled. Sternum dark brown, with abundant patches. Legs measurements: I 3.69 (1.17, 0.45, 0.82, 0.58, 0.67); II 3.4 (1.11, 0.64, 0.49, 0.6, 0.56); III 3.78 (1.27, 0.66, 0.5, 0.73, 0.62); IV 3.99 (1.21, 0.61, 0.68, 0.87, 0.62); spination (Fig. 4A, B): I Fe: d 1-1-1, pr0-0-2, rt0-0-2; Pa pr1-0-1, rt1-0-1; II Fe d1-1-1, pr 0-0-2, rt 0-0-2; Pa pr1-0-1, rt1-0-1; Ti pv1-1-1, rv1-1-1, pr0-1-0, rt0-1-0, d1-1-0; Met pv1-0-1, rv1-0-1, pr1-0-1, rt1-0-1; III Fe d1-1-0, pr0-0-2, rt0-0-2; Pa pr1-0-1, rt1-0-1; Ti pv1-0-1, rv1-0-1, pr1-1-0, rt1-1-0; Met pr1-0-1, rt1-0-1, pv1-0-1, rv1-0-1; IV Fe d1-1-1, pr0-0-1; Pa pr1-0-1, rt1-0-1; Ti pv1-0-1, rv1-0-1, pr1-1-0, rt1-1-0; Met pr1-1-1, rt1-1-1, pv1-0-1, rv1-0-1. Abdomen (Fig. 4A, B) ovoid, 2 long, 2.05 wide, yellowish to black-brown, with four horizontal stripes and a broad longitudinal stripe dorsally; venter brown.

***Epigyne*** (Fig. 4C, D). Copulatory openings located at antero-medial part of atrium. Copulatory ducts very long, convoluted, double swan-like in shape in ventral view, medially touching in dorsal view. Spermathecae round, medially located, separated by the touching copulatory ducts. Fertilization ducts slightly shorter than spermathecal length, directed laterally.

###### Distribution.

Known only from the type locality in Jiangxi Province, China (Fig. 13).

###### Etymology.

The species is named in honor of He Zizhen, another prominent figure associated with the hard and bitter struggle in Jinggangshan; noun in apposition.

###### Remarks.

This new species also closely resembles the *Neonwangi* Peng & Li, 2006; however, based on the spiral embolus, the slender copulatory duct, and the relatively small spermatheca, we considered the new species as belonging to the genus *Cytaea*. The species *N.wangi* within the genus *Neon* may be more closely related to the genus *Cytaea*.

#### ﻿Genus *Eupoa* Żabka, 1985

##### ﻿ Eupoa pengi

Taxon classificationAnimaliaAraneaeSalticidae

Liu, 2021

182F7B39-3E87-5A3B-8C72-B6F6C55AC937

Fig. 5

Eupoa pengi Liu, in Ying et al. 2021: 42, figs 1A–G, 2A–H (male holotype examined).

###### Material examined.

***Holotype*** ♂, China • Jiangxi Province: Ji’an County, Jinggangshan County Level City, Jinggang Mountain National Nature Reserve, Ciping Town, Xiaojing Village, Longtan Scenic Spot, 26°35'33.08"N, 114°8'18.50"E, 909 m a.s.l., 1 October 2018, Ke-ke Liu, Wen Sun and Hui-pu Luo leg. (Sal-019, ASM-JGSU).

###### Other material examined.

• 1 ♀, China • Jiangxi Province: Ji’an County, Jinggangshan County Level City, Maoping Town, Shenshan Village, Shenshan, 26°38'52.8"N, 114°6'21.6"E, 919 m a.s.l., 6 April 2014, Zhi-wu Chen, Yu-bao Tang, Ze-yuan Meng, Xiao-ping Huang and Ke-ke Liu leg. (Sal-019, ASM-JGSU); 1 ♀, 26°38'13.2"N, 114°6'39.6"E, 1099 m a.s.l., other data as same as previous (Sal-019, ASM-JGSU).

###### Diagnosis.

**Figure 5.**
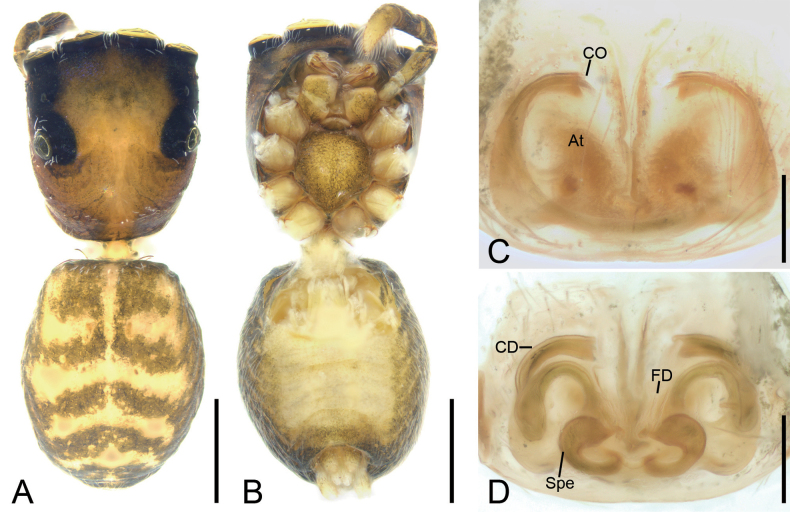
*Eupoapengi* Liu, 2021, female **A** habitus, dorsal view **B** same, ventral view **C** epigyne, ventral view **D** same, dorsal view. Abbreviations: At – atrium, CD – copulatory duct, CO – copulatory opening, FD – fertilization duct, Spe – spermatheca. Scale bars: 0.5 mm (**A, B**); 0.1 mm (**C**, **D**).

The female of this species can be easily distinguished from that of all other species by the nearly double C-shaped copulatory ducts (vs circular, one C-shaped, or other shapes in other species) and the C-shaped spermathecae (vs oval or spherical in other species) (Logunov and Marusik 2014; Peng 2020; Wang and Li 2022; Wang et al. 2023a) (Fig. 5C, D).

###### Description.

Habitus as in Fig. 5A, B. Total length 2.21, carapace yellow to brown, with large patches of black pigment around PME and PLE eye cups, 0.95 long, 0.77 wide, cup-shaped, with many short white setae in dorsal view. Eye sizes and interdistance (Fig. 5A): AME 0.24, ALE 0.14, PME 0.05, PLE 0.11, AME-AME 0.03, AME-ALE 0.01, PME-PME 0.58, PME-PLE 0.15, AME-PME 0.31, AME-PLE 0.48, ALE-ALE 0.52, PLE-PLE 0.59, ALE-PLE 0.32. MOA 0.61 long, front width 0.41, back width 0.73. Chelicerae (Fig. 5B) pale yellow, mottled, with two promarginal and four retromarginal teeth. Endites pale yellow, mottled, as long as wide. Labium yellow-brown, wider than long. Sternum (Fig. 5B) pale yellow-brown, mottled, broadly oval, slightly longer than wide, lateral margin slightly thickened, posterior end blunt. Abdomen (Fig. 5A, B) yellowish, dorsally with five pairs of dark brown stripes, anterior two touching, posterior three touching, 1.08 long, 0.91 wide, completely covered sparse elongated setae, anterior part with many discrete macrosetae; venter pale yellow. Legs missing.

***Epigyne*** (Fig. 5C, D). Atrium very large, oval, nearly covering all areas of epigyne; copulatory openings small, antero-medially located, separated by half width of atrium; copulatory ducts long, double C-shaped; spermathecae C-shaped, posterior part human stomach-shaped; fertilization ducts subpostero-medially located, slightly separated at base, directed anteriorly.

###### Distribution.

Known only from Jiangxi Province, China (Fig. 13).

###### Remarks.

Ying et al. (2021) first recognized the species as new and had labeled the male as a holotype, but did not recognize the other salticid specimens collected from nearby localities. This is probably because the (preserved) female is pale and presumably had faded in alcohol. After re-examination, these two female specimens were considered as allotypes of male *E.pengi*.

#### ﻿Genus *Orientattus* Caleb, 2020

##### ﻿ Orientattus chushu

Taxon classificationAnimaliaAraneaeSalticidae

K. Liu sp. nov.

AA951A93-FC3E-5ACA-A07A-E7B1AFABACC8

https://zoobank.org/B6BB6F83-9363-408C-9EA5-257615D0B98B

**Figure 6.**
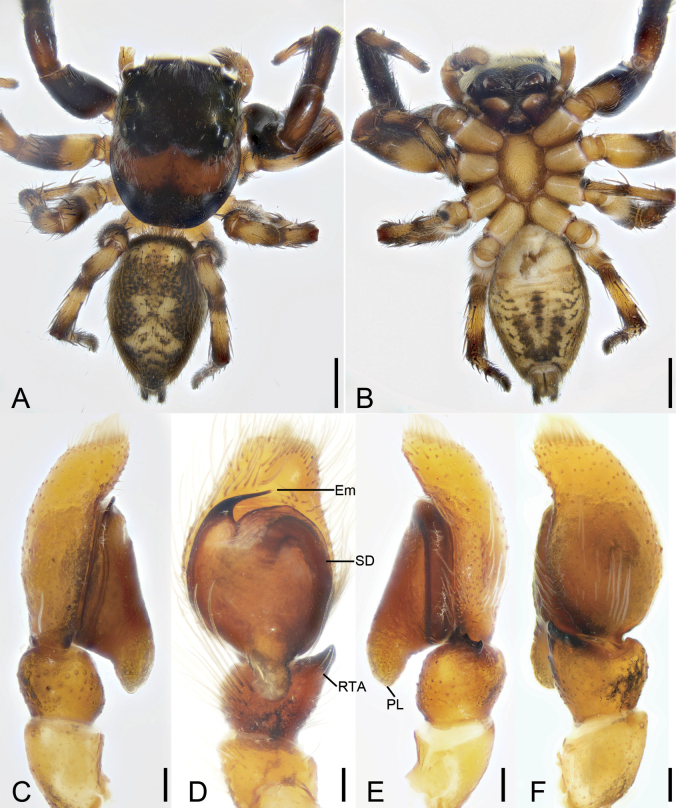
*Orientattuschushu* sp. nov., male holotype **A** habitus, dorsal view **B** same, ventral view **C** palp, prolatero-ventral view **D** same, ventral view **E** same, ventro-retrolateral view **F** same, dorsal view. Abbreviations: Em – embolus, PL – posterior lobe, RTA – retrolateral tibial apophysis, SD – sperm duct. Scale bars: 1 mm (**A**, **B**); 0.1 mm (**C**–**F**).

**Figure 7.**
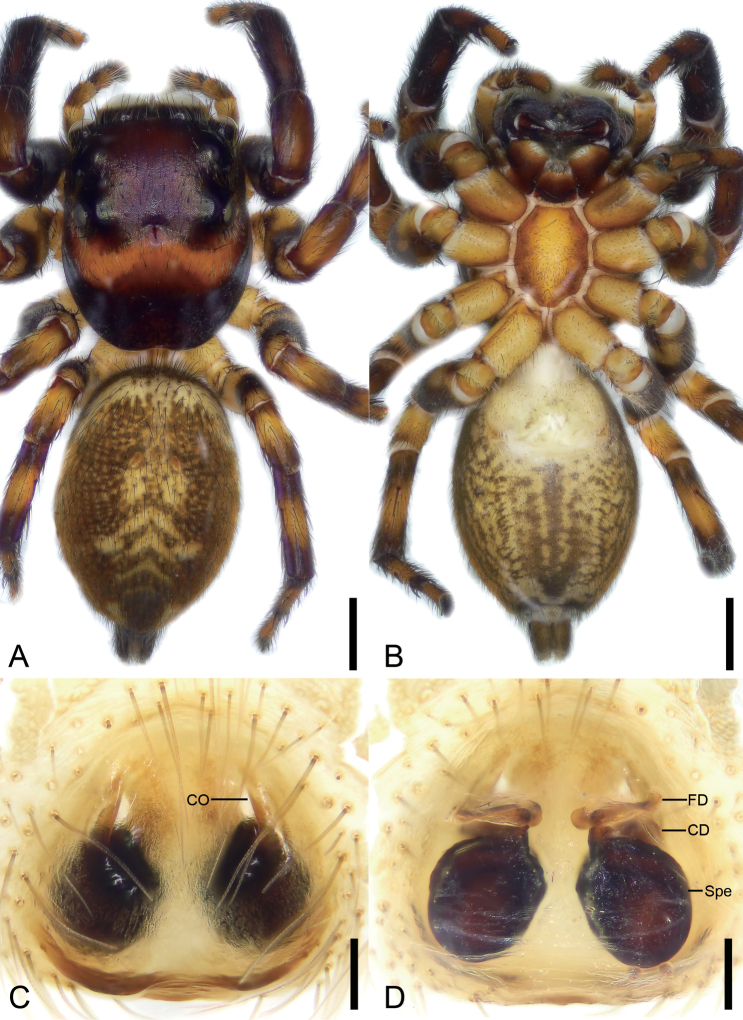
*Orientattuschushu* sp. nov., female paratype **A** habitus, dorsal view **B** same, ventral view **C** epigyne, ventral view **D** same, dorsal view. Abbreviations: CD – copulatory duct, CO – copulatory opening, FD – fertilization duct, Spe – spermatheca. Scale bars: 1 mm (**A, B**); 0.1 mm (**C**, **D**).

Figs 6 , 7 , 12B, C Common name: 楚舒东蛛

###### Type material.

***Holotype*** ♂, China • Jiangxi Province: Ji’an County, Jinggangshan County Level City, Ciping Town, Zhufeng Scenic Spot, 26°32'16.8"N, 114°8'38.4"E, 846 m a.s.l., 1 August 2014, Feng-bo Zhang, Yong-hong Xiao, Chu-shu Xie, Huo-kai Wang, Jiao-rong Liu, Ze-yuan Meng and Ke-ke Liu leg. (Sal-049, ASM-JGSU). ***Paratypes***: • 1 ♀, Xiaojing Village, Longtan Scenic Spot, 26°35'24"N, 114°8'9.6"E, 930 m a.s.l., 2 August 2014, other data as same as holotype (Sal-049, ASM-JGSU).

###### Diagnosis.

Male is similar to that of *Orientattusaurantius* (Kanesharatnam & Benjamin, 2018) (cf. Figs 6C−F, 12B, C and Caleb J. T. D. 2020: 2, figs 1–5) in having the rounded tegulum, same posterior lobe in ventral view and bifurcated retrolateral tibial apophysis, but can be easily distinguished from it by the longer curved embolus with a thin tip (vs the short embolus with a thick tip) and three forked RTA (vs two). The females of this species resemble *O.bowu* Lin, Wang & Ruan, 2024 (cf. Fig. 7C, D and Lin et al. 2024: 28, fig. 4A, B) in having a relatively small copulatory opening and a pair of pockets on the posterior margin of the epigynal plate, but can be separated by the closely spaced pockets (vs widely) and the oval-shaped spermathecae (vs reniform).

###### Description.

**Male (holotype).** Habitus (Fig. 6A, B). Total length 5.04. Carapace (Fig. 6A), anterior eyes area and posterior margin black, subposterior part red, length 2.36, width 1.88, with dense, short, white setae. Eyes (Fig. 6A) diameters and interdistances: AME 0.51, ALE 0.25, PME 0.18, PLE 0.28; AME–AME 0.13, ALE−AME 0.12, PME–PME 1.5, PLE−PME 0.16, AME−PME 0.76, AME−PLE 1.05, ALE−ALE 1.27, PLE−PLE 1.36, ALE−PLE 0.81. MOA 1.17 long, front width 1.15, back width 1.67. Chelicerae (Fig. 6B) black, straight, robust, with two retromarginal and one promarginal teeth. Endites brown, nearly as long as wide. Labium brown, wider than long. Sternum yellow to brown, longer than wide, with densely long setae. Legs yellow to black brown (Fig. 6A, B), with brown annulations; measurements: I 4.37 (1.23, 0.77, 1.14, 0.65, 0.58); II 4.16 (1.32, 0.69, 0.97, 0.59, 0.59); III 4.34 (1.34, 0.8, 0.85, 0.68, 0.67); IV 4.88 (1.5, 0.62, 1.06, 0.98, 0.72); spination: I Fe: d 1-0-0; Pa pr1-1-1; Ti pv1-1-1, rv1-1-1; Met pv1-0-1, rv1-0-1, v1-0-1; II Fe pr1-1-1; Pa pr1-1-2; Ti pv0-1-0, pr0-1-0; III Fe d1-1-1, pr0-0-1, rt0-0-1; Pa pr0-1-0; Ti pv0-1-1, rv0-0-1, pr0-1-1; Met pr1-0-0, rt1-0-1; IV Fe pr1-1-1, rt0-0-1; Pa pr0-0-1; Ti pv1-0-1, v0-0-1, pr1-0-0, rt1-0-0; Met pr1-1-0, rt1-1-1. Abdomen (Fig. 6A, B) elongate, oval, yellow to black-brown, with five yellow stripes, 2.61 long, 1.89 wide; venter yellow to brown, with U-shaped and straight serrated stripes.

***Palp*** (Figs 6C−F, 12B, C). Retrolateral tibial apophysis thumb-like in ventral view, apex directed ventrally, with three dentate processes in retrolateral view. Posterior lobe of tegulum elongated, nearly extending to the medial part of tibia. Sperm duct curved, arising from 12 o’clock. Embolus hook-shaped, arising from 10 o’clock, with a spine-like tip, directed retrolaterally.

**Female (paratype)**. As in male, except as noted. Habitus (Fig. 7A, B). Total length 7.23. Carapace (Fig. 7A) length 3.02, width 2.69. Eyes (Fig. 7A) diameters and interdistances: AME 0.58, ALE 0.37, PME 0.12, PLE 0.33; AME–AME 0.29, ALE−AME 0.27, PME–PME 1.87, PLE−PME 0.29, AME−PME 0.71, AME−PLE 1.04, ALE−ALE 1.72, PLE−PLE 1.98, ALE−PLE 0.72. MOA 1.04 long, front width 1.35, back width 2.12. Chelicerae (Fig. 7B) black, straight, robust, with two retromarginal and one promarginal teeth. Legs measurements: I−II missing; III 6.46 (2.02, 0.78, 1.52, 1.21, 0.93); IV 5.78 (2.05, 0.64, 1.32, 0.93, 0.84); spination (Fig. 7A, B): III Fe d1-0-1, pr0-0-1, rt0-0-1; Pa rv0-1-0; Ti pr1-0-1, rt1-0-1; Met pv1-0-1, rv1-0-1, pr1-0-1, rt1-0-1; IV d1-0-1, pr0-0-1, rt0-0-1; Ti pv1-0-1, rv1-0-1, pr1-0-1, rt1-0-1; Met missing. Abdomen (Fig. 7A, B) with seven yellow stripes from anterior to front of anus, 4.38 long, 3.27 wide.

***Epigyne*** (Fig. 7C, D). Copulatory openings located at antero-lateral part of epigyne, longitudinal, directed anteriorly. Copulatory ducts very short, curved. Spermathecae oval, anterior part closer than posterior. Fertilization ducts anteriorly located, directed laterally.

###### Distribution.

Known only from the Jiangxi Province locality (Fig. 13).

###### Etymology.

The specific name is a patronym for Miss Chushu Xie, who collected the specimens from Jingggang Mountain; noun in apposition.

###### Remark.

Due to the bifurcated RTA and the well-developed PL of this new species that closely resemble those of the type species *O.aurantius*, we place it in the genus *Orientattus*.

##### ﻿ Yaginumaella lobata

Taxon classificationAnimaliaAraneaeSalticidae

Peng, Tso & Li, 2002

CE1D0422-DF47-5DFF-BD8F-939A763FD673

Figs 8 , 9

Yaginumaella lobata Peng, Tso & Li, 2002: 6, figs 21−25 (male holotype examined); Peng 2020: 493, fig. 364a−e.

###### Additional material examined.

1 ♂, China • Jiangxi Province: Ji’an County, Jinggangshan County Level City, Jinggang Mountain National Nature Reserve, Ciping Town, Longtan Scenic Spot, 26°35'33.08"N, 114°8'18.50"E, 909 m a.s.l., 1 October 2014, Ke-ke Liu, Wen Sun and Hui-pu Luo leg. (Sal-019, ASM-JGSU); • 1 ♀, Xingzhou Village, Baimukeng, 26°31'4.8"N, 114°11'9.6"E, 669 m a.s.l., 3 October 2014, Jian-yun Wen, Tian-ming Wang, Lei Zhang, Ze-yuan Meng and Ke-ke Liu leg., other data as same as previous (Sal-289, ASM-JGSU); • 1 ♀, Luofu Town, Pingtou Village, Changguling Forest Farm, 26°38'52.8"N, 114°13'58.8"E, 524 m a.s.l., 5 October 2014, other data as same as previous (Sal-289, ASM-JGSU); • 2 ♀, Maoping Town, Shenshan Village, Shenshan, 26°38'52.8"N, 114°6'21.6"E, 919 m a.s.l., 6 April 2014, Zhi-wu Chen, Yu-bao Tang, Ze-yuan Meng, Xiao-ping Huang and Ke-ke Liu leg. (Sal-289, ASM-JGSU); • 1 ♀, 26°38'24"N, 114°6'39.6"E, 1029 m a.s.l., other data as same as previous (Sal-289, ASM-JGSU); • 1 ♀, Xiaping Town, 26°45'43.2"N, 114°16'12"E, 303 m a.s.l., Zhi-wu Chen, Ze-yuan Meng, Qian-qian Chen, Ke-ke Liu, Yi-fan Zhao and Sha Wu leg. (Sal-289, ASM-JGSU).

###### Diagnosis.

**Figure 8.**
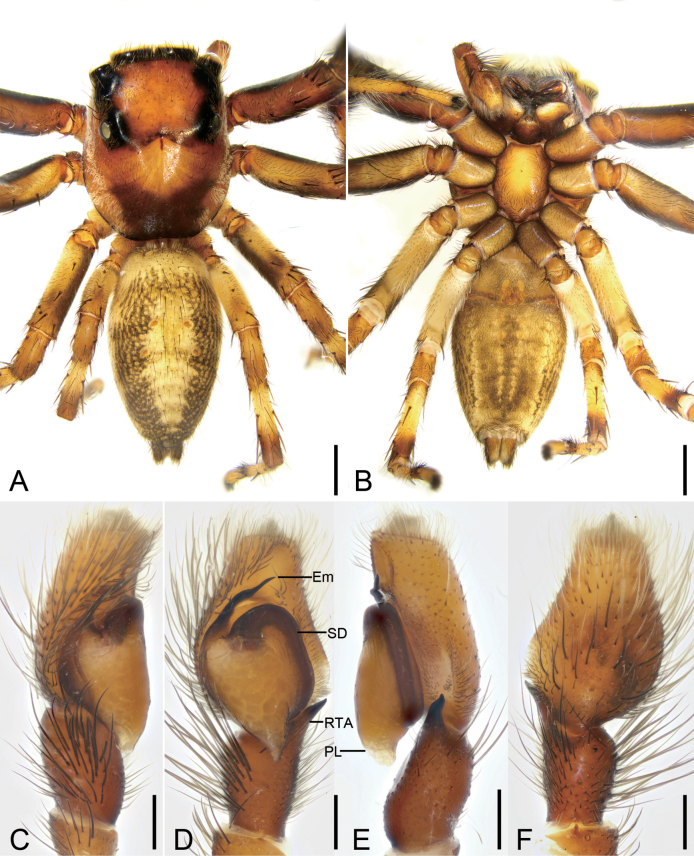
*Yaginumaellalobata* Peng, Tso & Li, 2002, male **A** habitus, dorsal view **B** same, ventral view **C** palp, prolatero-ventral view **D** same, ventral view **E** same, ventro-retrolateral view **F** same, dorsal view. Abbreviations: Em – embolus, PL – posterior lobe, RTA – retrolateral tibial apophysis, SD – sperm duct. Scale bars: 1 mm (**A**, **B**); 0.5 mm (**C**–**F**).

**Figure 9.**
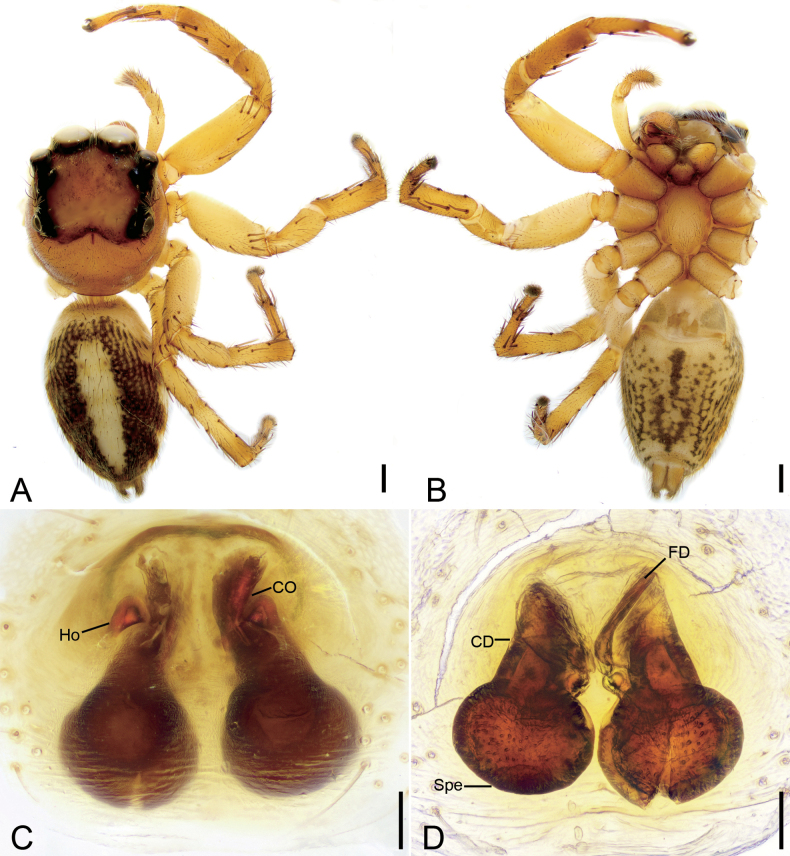
*Yaginumaellalobata* Peng, Tso & Li, 2002, female **A** habitus, dorsal view **B** same, ventral view **C** epigyne, ventral view **D** same, dorsal view. Abbreviations: CD – copulatory duct, CO – copulatory opening, FD – fertilization duct, Ho – hood, Spe – spermatheca. Scale bars: 0.5 mm (**A, B**); 0.1 mm (**C**, **D**).

Male is similar to that of *Yaginumaellaflexa* Song & Chai, 1992 (cf. Fig. 8C−F and Peng 2020: 492, fig. 363a-c) in having the long posterior lobe reaching 1/2 length of tibia and curved sperm duct arising from 9 o’clock, but can be easily distinguished from it by the horn-like retrolateral tibial apophysis (vs nearly C-shaped in *Y.flexa*) and the relatively long embolus with an uneven and thin spine-like tip (vs spine-like, tapering in *Y.flexa*). The female of this species resembles that of *Y.longnanensis* Yang, Tang & Kim, 1997 (cf. Fig. 9C, D and Peng 2020: 494, fig. 365d, e) in having the short but broad copulatory ducts and the nearly round spermathecae, but can be easily distinguished from it by the sloping extending copulatory ducts (vs parallel in *Y.longnanensis*) and the spermathecae separated from each other (vs touching in *Y.longnanensis*).

###### Description.

Male. Habitus (Fig. 8A, B). Total length 7.84. Carapace (Fig. 8A) yellow to brown, posteriorly with the broad V-shaped brown mark, lateral margin covered by abundant white feathery setae, length 3.81, width 3.05. Eyes (Fig. 8A) diameters and interdistances: AME 0.79, ALE 0.48, PME 0.08, PLE 0.40; AME–AME 0.25, ALE−AME 0.23, PME–PME 2.18, PLE−PME 0.43, AME−PME 1.03, AME−PLE 1.59, ALE−ALE 1.94, PLE−PLE 2.08, ALE−PLE 1.16. MOA 1.5 long, front width 1.72, back width 2.38. Chelicerae (Fig. 8B) yellow-brown, with two retromarginal teeth and one retromarginal tooth. Endites yellow-brown, nearly as long as wide. Labium brown, slightly wider than long. Sternum yellow-brown, longer than wide, with dense long setae. Legs yellow, with brown annulations (Fig. 8A, B); measurements: I 5.78 (1.56, 1.04, 1.82, 0.79, 0.57); II 5.23 (1.53, 0.7, 1.38, 0.99, 0.63); III 6.55 (1.65, 1.41, 1.52, 1.3, 0.67); IV 5.77 (1.4, 1.06, 1.45, 1.21, 0.65); spination: I Fe d 1-1-1, pr 0-0-1, rt 0-0-2; Ti pv 1-2-1, rv 1-2-1; Met pv 1-0-1, rv 1-0-1; II Fe d 0-2-0, pr 0-0-2, rt 0-0-2; Ti pr 1-1-0, rt 1-1-0, pv 1-1-1, rv 1-1-1; Met pv 1-0-0, rv 1-0-0, pr 1-0-1, rt 1-0-1; III Fe d 1-1-1, pr 0-0-1, rt 0-1-2; Pa pr 1-1-0, rt 1-1-0; Ti pr 1-1-1, rt 1-1-1, pv 0-0-1, rv 1-0-1; Met pr 1-0-1, rt 1-0-1, pv 1-0-1, rv 1-0-1; IV Fe d 1-1-0, pr 0-0-1, rt 0-0-2; Pa pr 1-1-0 rt 1-1-0; Ti pv 0-1-0, rv 0-1-0, pr 1-1-1, rt 1-1-1; Met pr 1-0-1, rt 1-0-1, pv 1-0-1, rv 1-0-1. Abdomen (Fig. 8A, B) yellowish to dark yellow-brown, medially with yellow serrated stripe, laterally with many spots; venter yellow to brown, medially with a trident-shaped stripe, 3.91 long, 2.38 wide.

***Palp*** (Fig. 8C−F). Retrolateral tibial apophysis thick, finger-like, directed dorsally in ventral view, nearly as long as 1/2 of tibia length, with very short, slightly curved apex. Sperm duct curved, arising from ~ 10 o’clock. Posterior lobe of tegulum elongated, nearly extending to 1/2 of tibia length. Embolus shorter than width of tegulum, gradually tapering to a point, with the spine-like apex of uneven thickness, ending at ~ 12 o’clock.

**Female**. Habitus (Fig. 9A, B). As in male, except as noted. Total length 7.47. Carapace (Fig. 9A) dark red-brown, length 3.59, width 2.96. Eyes (Fig. 9A) diameters and interdistances: AME 0.68, ALE 0.39, PME 0.12, PLE 0.38; AME–AME 0.18, ALE−AME 0.29, PME–PME 2.04, PLE−PME 0.42, AME−PME 0.91, AME−PLE 1.47, ALE−ALE 1.74, PLE−PLE 1.91, ALE−PLE 1.07. MOA 1.38 long, front width 1.59, back width 2.18. Chelicerae and endites yellow, with two retromarginal teeth and one retromarginal tooth. Labium red-brown. Sternum yellow, with red margin. Legs measurements: I 7.05 (2.19, 1.37, 1.55, 0.99, 0.95); II 5.42 (1.7, 0.98, 1.4, 0.68, 0.66); III 7.8 (2.81, 1.29, 1.25, 1.45, 1); IV 7.68 (2.57, 1.5, 1, 1.53, 1.08); spination (Fig. 9A, B): I Fe: d 1-1-1, pr0-0-3, rt0-0-2; Pa pr0-1-0, rt0-1-0; Ti pv1-2-1, rv1-2-1, rt1-0-0; Met pv1-0-1, rv1-0-1; II Fe d1-1-1, pr 0-0-3, rt 0-0-2; Pa pr0-1-0, rt0-1-0; Ti pv1-1-2, rv1-1-2, rt1-1-0; Met pv1-0-1, rv1-0-1; III Fe d1-1-1, pr0-0-3, rt0-0-2; Pa pr0-1-0, rt0-1-0; Ti pv1-1-1, rv0-1-0, pr1-1-0, rt1-1-0; Met pr1-0-1, rt1-0-1, pv1-0-1, rv1-0-1; IV Fe d1-1-1, pr0-0-2; Pa pr0-1-0, rt0-1-0; Ti pv1-0-1, rv0-1-0, pr1-1-1, rt1-1-1; Met pr1-1-1, rt1-1-1. Abdomen (Fig. 9A, B) ovoid, 3.78 long, 2.38 wide; venter with a clavate brown stripe medially and nearly U-shaped brown stripes posteromedially.

***Epigyne*** (Fig. 9C, D). Copulatory openings located at postero-lateral part of atrium, covered by the nearly C-shaped margin, accompanied with the triangular hoods. Copulatory ducts short, slanted posteriorly in dorsal view. Spermathecae round, barely touching each other. Fertilization ducts slightly longer than copulatory ducts, medially located at anterior spermathecae.

###### Distribution.

Known only from the type locality in Taiwan and Jiangxi Province, China (Fig. 13).

###### Remarks.

According to Peng et al. (2002) the original material consisted of two male specimens; the paratype was recorded from Jiangxi province, but the details of the type locality were omitted and not provided in their paper. The male and female specimens of this species are, unsurprisingly, recollected from Jinggang Mountain National Nature Reserve in the same province.

#### ﻿*Yaginumanis* Wanless, 1984

##### ﻿ Yaginumanis yuanwencai

Taxon classificationAnimaliaAraneaeSalticidae

K. Liu sp. nov.

675D3ABC-3D9D-5394-9FE4-8210B62A77DB

https://zoobank.org/9EE40518-BA53-4AAF-A293-B2D824B57090

**Figure 10.**
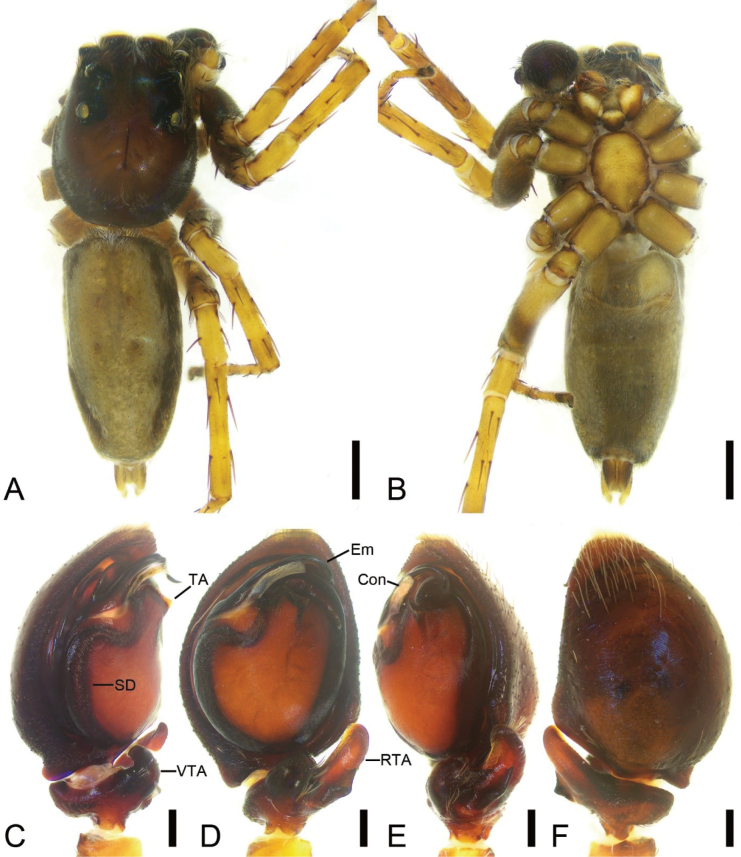
*Yaginumanisyuanwencai* sp. nov., male holotype **A** habitus, dorsal view **B** same, ventral view **C** palp, prolatero-ventral view **D** same, ventral view **E** same, ventro-retrolateral view **F** same, dorsal view. Abbreviations: Con – conductor, Em – embolus, RTA – retrolateral tibial apophysis, SD – sperm duct, TA – tegular apophysis, VTA – ventral tibial apophysis. Scale bars: 0.5 mm (**A**, **B**); 0.2 mm (**C**–**F**).

**Figure 11.**
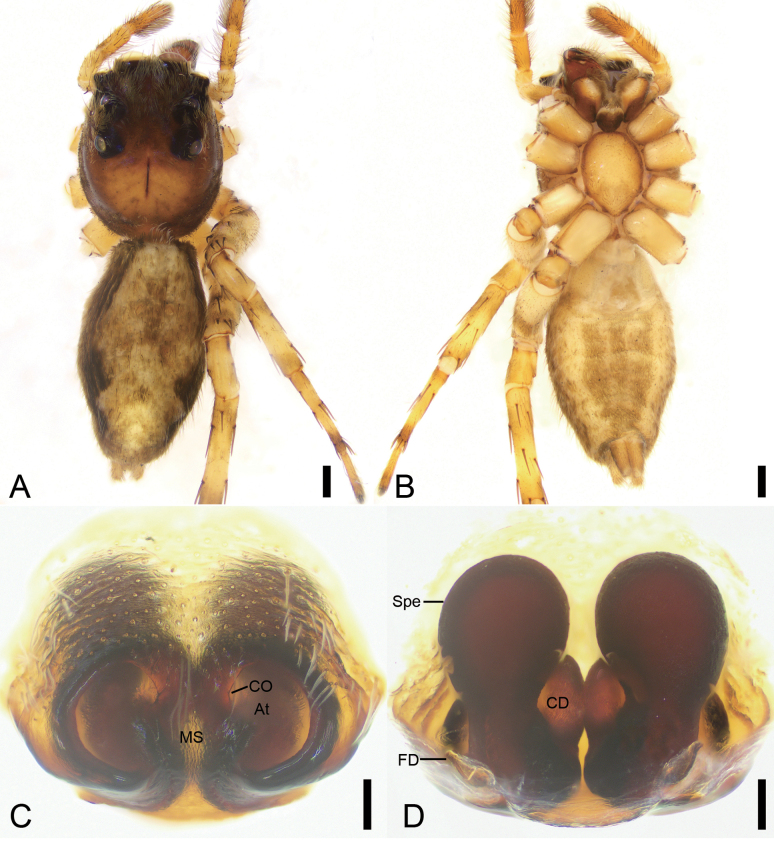
*Yaginumanisyuanwencai* sp. nov., female paratype **A** habitus, dorsal view **B** same, ventral view **C** epigyne, ventral view **D** same, dorsal view. Abbreviations: At – atrium, CD – copulatory duct, CO – copulatory opening, FD – fertilization duct, MS – median septum, Spe – spermatheca. Scale bars: 0.5 mm (**A, B**); 0.1 mm (**C**, **D**).

**Figure 12.**
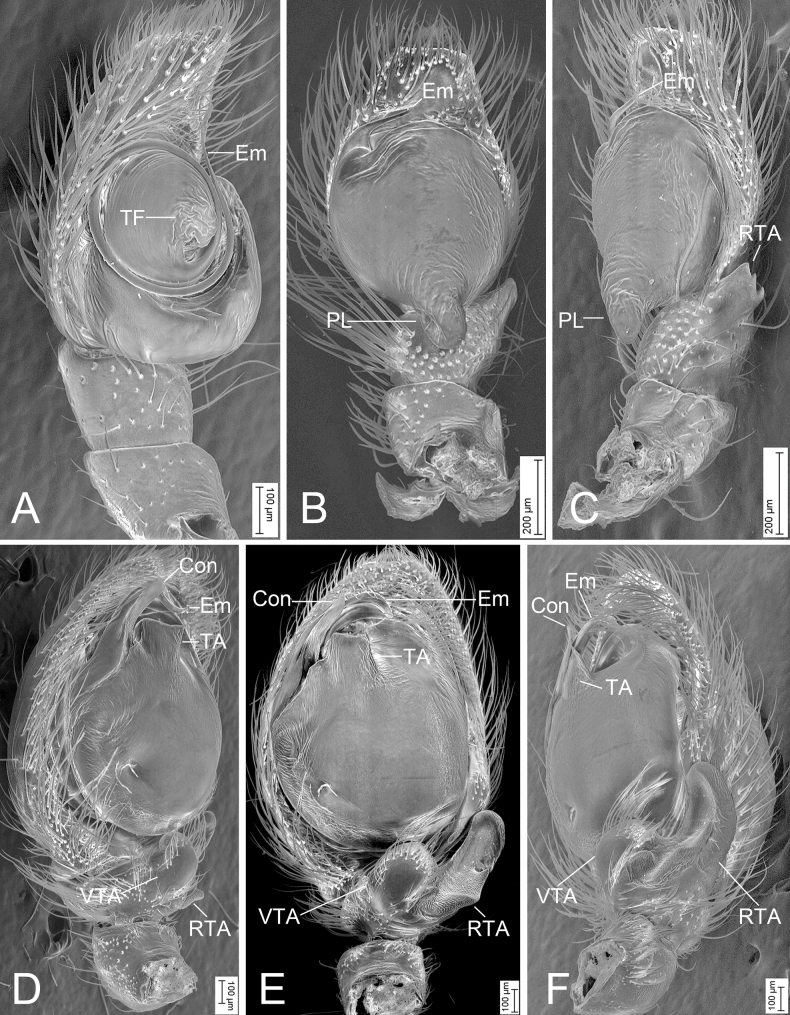
SEMs of male palps **A***Charippuswangzuo* sp. nov., male holotype, ventro-prolateral view **B***Orientattuschushu* sp. nov., male holotype, ventral view **C** same, ventro-retrolateral view **D***Yaginumanisyuanwencai* sp. nov., male holotype, ventro-prolateral view **E** same, ventral view **F** same, retrolatero-ventral view. Abbreviations: Con – conductor, Em – embolus, PL – posterior lobe, RTA – retrolateral tibial apophysis, TA – tegular apophysis, TF – tegular flap, VTA – ventral tibial apophysis.

Figs 10 , 11 , 12D–F Common name: 袁文才后雅蛛

###### Type material.

***Holotype*** ♂, China • Jiangxi Province: Ji’an County, Jinggangshan County Level City, Jinggang Mountain National Nature Reserve, Ciping Town, Longtan Scenic Spot, 26°35'24"N, 114°8'16.8"E, 939 m a.s.l., 1 June 2014, Ke-ke Liu, Yu-bao Tang, Ze-yuan Meng, Xiao-ping Huang, Zhi-wu Chen, and Zhan-feng Wang leg. (Sal-097, ASM-JGSU). ***Paratypes***: • 1 ♀, Dajing Village, Dajing Forest Farm, 26°34'12"N, 114°7'19.2"E, 956 m a.s.l., 27 August 2015, Zhi-wu Chen, Chun-qi Dong, Ji-hao Zhang, etc. leg., other data as same as holotype (Sal-171, ASM-JGSU); • 1 ♀, Dajing Village, Jiangjun Park, 26°33'54"N, 114°7'30"E, 927 m a.s.l., 26 August 2015, other data as same as previous data (Sal-171, ASM-JGSU); • 2 ♀, Luofu Town, Xiangzhou Village, 26°37'8.4"N, 114°16'12"E, 922 m a.s.l., 13 July 2015, Ce Xu, Shi-cong He, Ze-yuan Meng, Sha Wu, Yi-fan Zhao and Ke-ke Liu leg., other data as same as previous data (Sal-171, ASM-JGSU).

###### Diagnosis.

Male is similar to that of *Yaginumanissexdentatus* (Yaginuma, 1967) (cf. Fig. 10C−F and Bohdanowicz and Prószyński 1987: 46, figs 1–3) in having the fan-shaped ventral tibial apophysis and finger-like retrolateral tibial apophysis with a curved basal apophysis, but can be easily distinguished from it by the broad strongly curved embolus (vs relatively thin and only slightly curved in *Y.sexdentatus*), the band-like conductor (vs oval in *Y.sexdentatus*), and the nearly square tegular apophysis (vs triangular in *Y.sexdentatus*) in ventral view. The female of this species resembles that of *Y.sexdentatus* (cf. Fig. 11C, D and Bohdanowicz and Prószyński 1987: 46, figs 4, 5) in having the spermathecae with a constriction medially, but can be easily distinguished from it by the large atrium covering more than 1/2 of the epigynal plate (vs small, < 1/3 of epigynal plate in *Y.sexdentatus*), and the distinct broad copulatory ducts (vs indistinct in *Y.sexdentatus*). The female of this species also resembles that of *Y.wanlessi* Zhang & Li, 2005 (cf. Fig. 11C, D and Zhang and Li 2005: 227, fig. 5D, E) in having a thickened arc-shaped margin of the copulatory openings, but can be easily differentiated by the spermathecae having a distinct constriction (vs absent in *Y.wanlessi*).

###### Description.

**Male (holotype).** Habitus (Fig. 10A, B). Total length 7.56. Carapace (Fig. 10A) brown to dark, medially with a slender dark fovea, the broad V-shaped brown mark, lateral and posterior margins covered by abundant white feathery setae, length 3.45, width 2.61. Eyes (Fig. 10A) diameters and interdistances: AME 0.56, ALE 0.32, PME 0.23, PLE 0.29; AME–AME 0.19, ALE−AME 0.21, PME–PME 1.39, PLE−PME 0.39, AME−PME 0.30, AME−PLE 0.99, ALE−ALE 1.37, PLE−PLE 1.43, ALE−PLE 0.79. MOA 0.93 long, front width 1.18, back width 1.72. Chelicerae (Fig. 10B) yellow to brown, with three retromarginal teeth and five retromarginal tooth. Endites yellow, longer than wide. Labium yellow to brown, wider than long. Sternum yellow to brown, longer than wide, with dense long setae, laterally with three sclerotized serration-shaped extensions directed at coxae I−III. Legs (Fig. 10A, B) yellow, with brown annulations on femora; measurements: I 8.13 (2.33, 1.1, 1.99, 1.83, 0.88); II 7.77 (2.34, 1, 1.68, 1.99, 0.76); III 7.4 (2.27, 0.99, 1.77, 1.86, 0.51); IV 10.48 (2.84, 1.12, 2.46, 2.98, 1.08); spination: I Fe d 1-1-1, pr 0-0-2, rt 0-0-2; Pa pr 1-0-1, rt 1-0-1; Ti d 1-1-0, pr 1-0-1, rt 1-0-1; pv 1-1-1, rv 1-1-1; Met d 1-0-1, pv 1-1-1, rv 1-1-1; II Fe d 1-1-1, pr 0-0-2, rt 0-0-2; Pa pr 1-0-1, rt 1-0-1; Ti d 1-1-0, pr 1-0-1, rt 1-0-1, pv 1-1-1, rv 1-1-1; Met pr 1-1-1, rt 1-1-1, pv 1-0-1, rv 1-0-1; III Fe d 1-1-1, pr 0-1-2, rt 0-1-2; Pa pr 1-0-1, rt 1-0-1; Ti d 1-0-1, pr 1-0-1, rt 1-0-1, pv 1-1-1, rv 1-1-1; Met pr 1-1-1, rt 1-1-1, pv 1-0-1, rv 1-0-1; IV Fe d 1-1-1, pr 0-0-2, rt 0-0-2; Pa pr 1-0-1, rt 1-0-1; Ti d 1-0-1, pr 1-0-1, rt 1-0-1, pv 1-1-1, rv 1-1-1; Met pr 1-1-1, rt 1-1-1, pv 1-0-1, rv 1-0-1. Abdomen (Fig. 10A, B) yellow to dark brown, medially with pale band, laterally with dense black setae; venter yellow to brown, medially with a broad stripe, 4.08 long, 2.26 wide.

***Palp*** (Figs 10C−F, 12D−F). Ventral tibial apophysis thick, fan-shaped in ventral view, as long as tibia. Retrolateral tibial apophysis thick, finger-like, nearly 2× longer than ventral tibial apophysis, with a large triangular basal apophysis and a blunt tip in ventral view. Sperm duct curved, arising from ~ 10 o’clock. Tegular apophysis triangular in ventro-retrolateral view, n-shaped in ventro-prolateral view. Conductor membranous, band-shaped, shorter than half of embolus. Embolus thick, longer than tegular length.

**Female (paratype).** Habitus (Fig. 11A, B). As in male, except as noted. Total length 7.08. Carapace (Fig. 11A), length 3.2, width 2.46. Eyes (Fig. 11A) diameters and interdistances: AME 0.49, ALE 0.3, PME 0.28, PLE 0.29; AME–AME 0.23, ALE−AME 0.24, PME–PME 1.35, PLE−PME 0.43, AME−PME 0.55, AME−PLE 1.31, ALE−ALE 1.41, PLE−PLE 1.43, ALE−PLE 1. MOA 1.07 long, front width 1.18, back width 1.72. Chelicerae (Fig. 11B) and endites with three retromarginal teeth and six retromarginal tooth. Sternum yellow, laterally with two sclerotized serration shaped extensions directed at coxae I−II. Legs measurements: I 7.08 (2.14, 1.14, 1.59, 1.39, 0.82); II 6.83 (2.13, 1.15, 1.49, 1.25, 0.81); III 6.71 (1.97, 0.95, 1.48, 1.4, 0.91); IV 8.89 (2.44, 1.01, 2.25, 2.29, 0.9); spination (Fig. 11A, B): I Fe d 1-1-1, pr 0-0-2, rt 0-0-1; Pa pr 0-1-0; Ti d 1-0-1, pv 1-1-1, rv 1-1-1; Met pv 1-1-1, rv 1-1-1; II Fe d 1-1-1, pr 0-0-2, rt 0-0-2; Pa pr 0-0-1, rt 0-0-1; Ti pr 1-0-1, pv 1-1-1, rv 1-1-1; Met pv 1-1-1, rv 1-1-1; III Fe d 1-1-1, pr 1-0-2, rt 1-0-2; Pa pr 0-1-0, rt 0-1-0; Ti d 1-0-0, pr 1-0-1, rt 1-0-1, pv 1-1-1, rv 1-1-1; Met pr 1-1-1, rt 1-1-1, pv 1-0-1, rv 1-0-1; IV Fe d 1-1-1, pr 1-0-1, rt 1-0-1; Pa pr 0-1-0, rt 0-1-0; Ti d 1-0-0, pr 1-0-1, rt 1-0-1, pv 1-1-1, rv 1-1-1; Met pr 1-1-1, rt 1-1-1, pv 1-0-1, rv 1-0-1. Abdomen (Fig. 11A, B) 3.88 long, 2.23 wide; venter with a trident-shaped stripe.

***Epigyne*** (Fig. 11C, D). Atrium oval, separated by a median septum, with a thickened margin. Copulatory openings oval, located at the antero-lateral part of atrium. Copulatory ducts short, touching. Spermathecae bowling-ball-like, slightly separated. Fertilization ducts short, directed anterolaterally.

###### Distribution.

**Figure 13.**
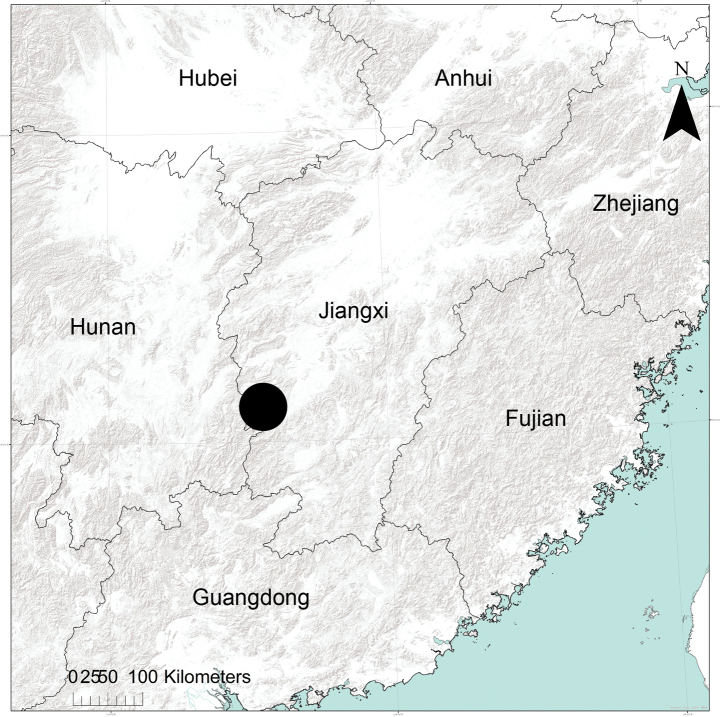
The location of the Jinggangshan National Nature Reserve in China indicated by the large black dot.

Known only from the type locality in Jiangxi Province, China (Fig. 13).

###### Etymology.

The species is named in honor of Yuan Wencai, also referring one of the prominent figures in the struggle in Jinggangshan; noun in apposition.

## ﻿Discussion

Since there is such an abundance of spider species as well as other organisms in this mountain, it is understandable that the nature reserve was selected to be included in the World Network of Biosphere Reserves. Several conclusions can be drawn from the morphological results based on the spider specimens collected from Jinggang Mountain National Nature Reserve in the past 12 years. Forty-five species are only known from a single sex: 28 of these were described from females and 17 from males (nearly half the number of females). The differences may be caused by a number of factors. First, males are preyed upon by predators during mate-searching (Gaskett et al. 2004). Second, cannibalistic behavior is present in some female species after mating (Dean and Sterling 1985). Third, the probability of dying is more likely between male rivals (Dodson and Beck 1993). Fourth, the samples in this study were collected during different seasons and months. And finally, the females usually inhabit their nests which are built in well-hidden places such as cavities on the underside of stones, crevices, and bark of various trees (Guseinov et al. 2004), resulting in more easy collection by many methods. As a result, the longevity of females compared to males is an important factor, and some female salticid species can live up to 3× longer than their male counterparts (Guseinov et al. 2004).

Research in China of salticid spider diversity is rather sparse, except for few taxonomic works. In 2016, Wang reported 78 species in 42 genera based on investigations and research from Huping Mountain National Nature Reserve, Hunan Province (Wang 2016). Surprisingly, a maximum of 114 species in 45 genera of these jumping spiders was recorded from Wuling Mountains (Liu 2020). Wang (2022) subsequently listed 157 species of Salticidae described from 62 genera from the Yunnan-Guizhou Plateau. In this study, we found that there are so many salticid species in this one place alone, and suspect that the true species richness of the salticid community might be much more than we have documented. Additionally, more accurate data based on the systematical surveys will be necessary for revealing the true salticid spider diversity from this province in the near future. All of these records suggest that there are still many jumping spider species unknown from China.

## Supplementary Material

XML Treatment for Charippus wangzuo

XML Treatment for Cytaea hezizhen

XML Treatment for Eupoa pengi

XML Treatment for Orientattus chushu

XML Treatment for Yaginumaella lobata

XML Treatment for Yaginumanis yuanwencai

## ﻿Additional information

### Author ORCIDs

Bin-lu Liu https://orcid.org/0009-0009-3080-8987

Yan-bin Yao https://orcid.org/0000-0002-2560-9299

Zi-han Cai https://orcid.org/0009-0005-5485-9296

Zhong-jing Wang https://orcid.org/0009-0000-6377-6840

Ke-ke Liu https://orcid.org/0000-0001-7822-3667

### Data availability

All of the data that support the findings of this study are available in the main text.
